# A review of spatial characteristics influencing circular economy in the built environment

**DOI:** 10.1007/s11356-023-26326-5

**Published:** 2023-03-17

**Authors:** Ning Zhang, Karin Gruhler, Georg Schiller

**Affiliations:** Leibniz Institute of Ecological Urban and Regional Development (IOER), Weberplatz 1, 01217 Dresden, Germany

**Keywords:** Circular economy, Built environment, Space, Construction materials, Circularity, Literature review

## Abstract

Industrialization, population growth, and urbanization are all trends driving the explosive growth of the construction industry. Creating buildings to house people and operate industry, together with building infrastructure to provide public services, requires prodigious quantities of energy and materials. Most of these virgin materials are non-renewable, and resource shortages caused by the development of the built environment are becoming increasingly inevitable. The gradually evolved circular economy (CE) is considered a way to ease the depletion of resources by extending service life, increasing efficiency, and converting waste into resources. However, the circularity of construction materials shows heavy regional distinctness due to the difference in spatial contexts in the geographical sense, resulting in the same CE business models (CEBMs) not being adapted to all regions. To optimize resource loops and formulate effective CEBMs, it is essential to understand the relationship between space and CE in the built environment. This paper reviews existing publications to summarize the research trends, examine how spatial features are reflected in the circularity of materials, and identify connections between spatial and CE clues. We found that the majority of contributors in this interdisciplinary field are from countries with middle to high levels of urbanization. Further, the case analysis details the material dynamics in different spatial contexts and links space and material cycles. The results indicate that the spatial characteristics can indeed influence the circularity of materials through varying resource cycling patterns. By utilizing spatial information wisely can help design locally adapted CEBMs and maximize the value chain of construction materials.

## Introduction

Significant demand for natural resources has arisen with the massive expansion of the cities and the rising population worldwide. The development of the built environment is the largest consumer of resources, consuming approximately 35–45% of materials and contributing 40% of global GHG emissions associated with material use (Hertwich et al. 2020; Mhatre et al. 2021). The ensuing resource exploration and related environmental impacts have intensified. It is estimated that the global consumption of building materials has tripled from 2000 to 2017 and produced 30–40% of the world’s solid waste and nearly 5 Gt CO_2_ emissions, or 10% of global annual emissions (EMF 2015; Pomponi and Moncaster 2017; Hertwich et al. 2020; López Ruiz et al. 2020; Huang et al. 2020).

The built environment is the physical surroundings created by humans for activities, ranging from personal places to large-scale urban settlements that often include buildings, cultural landscapes, and their supporting infrastructure (Moffatt and Kohler 2008; Hollnagel 2014). Opoku (2015) points out that the built environment is not only the physical environment but also the interaction of people in the local community and their cultural experiences. The physical constituents of which differ significantly from other products in that they are characterized by long lifetimes, numerous stakeholders, and hundreds of components and ancillary materials interacting dynamically in the spatial and temporal dimensions (Hart et al. 2019). The inherent complexity within the built environment is seen as a challenge for sustainable urban transition (Pomponi and Moncaster 2017).

Circular economy (CE) is one of the essential conditions and solutions for fostering and promoting sustainability (Geissdoerfer et al. 2017). The CE is an economic or industrial concept that distinguishes itself from the traditional linear economy of unsustainability. It is often understood as a restorative and regenerative economic model that includes three types of business models (CE business models/CEBMs): (1) those that increase resource efficiency and reduce resource consumption (narrowing); (2) those that promote reuse and extended service life through repair, remanufacture, upgrades and retrofits (slowing); and (3) those that convert waste into resources by recycling materials (closing) (Stahel 2016; Kirchherr et al. 2017; Figge et al. 2018; Geisendorf and Pietrulla 2018; Gallego-Schmid et al. 2020). It is also well known that urban systems often exhibit linear material flows and inefficient use of resources (Huang and Hsu 2003). Turning linear practices into circularity and maximizing the utility and value of resources is becoming a new model for production and consumption to protect the environment, mitigate climate change, and conserve resources (Cheshire 2019; Harris et al. 2021; Zeng et al. 2022). But incorrect policy formulation and thoughtless pursuit of CE strategies can negatively affect (Corvellec et al. 2021). Many voices currently argue that CE lacks any actual consensus on the magnitude of the economic, social, and environmental “win–win-win” benefits (Aguilar-Hernandez et al. 2021) and even leads to more significant environmental impacts, economic unsuccess, and employment losses (Spoerri et al. 2009; Schröder et al. 2020; Blum et al. 2020).

Circularity in the built environment refers to an approximation in terms of the materiality of immobile elements of the built environment, such as buildings and infrastructures, and their dynamics. These elements are predominantly composed of bulk building materials, mainly non-metallic mineral materials (Schiller et al. 2017b; Gontia et al. 2018; Yang et al. 2020). Despite few products are manufactured, purchased, disposed of, and recycled in the same geographic location in today’s global market (Skene 2018), the transportation distances of these bulk building materials are limited compared to other types of products due to their low specific value-added (Schiller et al. 2017a). Therefore, Schiller et al. (2017a) point out that analyses on (also circular) material flow in the built environment should be applied regionally, which also applies to studies of the availability and security of the supply of natural raw materials in the built environment (Schiller et al. 2020). It can be concluded that the regional context or the spatial context in the geographical sense (Scholl et al. 1996), in which the built environment is integrated, has a decisive influence on material flows in general and their circularity in particular.

Space is a central concept in geography that broadly consists of two distinctive interpretations: a fundamental attribute of reality (often used with time) and a counting term that denotes human conceptual constructs borne of individual experience and societal factors (Newell and Cousins 2015; Grossner 2017). Spatiality and space are two frequently confused concepts. In contrast to space, spatiality is spatial practices rather than an exogenously given and absolute coordinate system that refers to the ongoing processes and imaginations of making space/materials, regulating behaviors, and creating experiences (Mayhew 2015; Kobayashi 2017). Space is a more relevant core term than spatiality in discussing the built environment in the physical sense rather than the formation process. The importance of space in the circularity of the built environment has been implicitly mentioned in many studies on spatial structure and land use planning (Remøy et al. 2019; Lanau and Liu 2020; Gallego-Schmid et al. 2020). Additional studies have also provided fragmented evidence on characteristics of spatial distribution patterns in the built environment that impact the circular flow of materials (e.g., residential and housing density) (Condeixa et al. 2017).

Previous studies on built environment management have only touched on the importance of space aspects in a fragmented and implicit manner, leaving the specific relationship between space and CE unclear. This lack of clarity can negatively impact the development of CE strategies and the design of CEBMs. The research aims to fill this gap by systematically organizing and examining the relation between space and CE in the built environment. As a result, the specific purposes of this study are to determine through quantitative and qualitative literature review (1) which spatial factors can influence the circularity of materials in the built environment; (2) in which way they act on the material cycles; and (3) how spatial information can be systematized to serve the regionally adapted selection of CE strategies and design of CEBMs. These targets were achieved by combining a literature review with regional case analysis that discussed circularity of construction materials under the four themes of “built environment,” “materiality,” “circular economy,” and “space” and structuring the fragmented information. The remainder of this paper is structured in four sections. "Methods and materials" presents the methodology applied to perform the literature review, followed by "Results" that gives the obtained results. Finally, a discussion of the results and main findings is presented in "Discussion", and in "Conclusion", key contributions are provided in the conclusions part.

## Methods and materials

### Terms and definitions

This paper focuses on spatial features associated with CE in the built environment. Although the three core terms of “built environment” (BE), “circular economy” (CE), and “space” are described in the introduction, we prefer to conceptualize the terms based on the context of this study rather than using generic concepts before conducting the subsequent sections.

#### Built environment

The built environment is defined as the artificial environment that provides the setting for human activity. But for sociologists, the built environment also has an implicit social meaning that includes human communities, cultural experiences, and human interactions in addition to buildings (Holm 2003; Pedersen Zari and Jenkin 2009; Opoku 2015). The focus of this study is on the physical composition of the built environment itself, i.e., the materials and resources in buildings and infrastructure.

#### Circular economy

There are multiple voices on the concept of CE, and Vermeșan et al. (2020) mentioned that the concept of CE should be in a dynamic process of metamorphosis and adaptation, both as regards its theoretical as well as its practical aspect. One of the most widely accepted definitions of CE is a regenerative economic model or production approach in which material and energy cycles are slowed, closed, and narrowed through durable design, maintenance, repair, reuse, refurbishment, recycling, and reduction to minimize resource inputs and waste, emissions, and energy leakage (Geissdoerfer et al. 2017; Nobre and Tavares 2021). In this paper, CE strategies for managing immobile resources (bulk materials) in the built environment can be understood as the management of the circularity of the constituent resources of buildings and infrastructure by optimizing and controlling the dynamics and stocks of resources. Therefore, the metric indicators for CE are not only recycling rate, but also stock availability, resource productivity, resource reusability, and material retention time (durability) (Velenturf et al. 2019; Parchomenko et al. 2019; García-Barragán et al. 2019; Moraga et al. 2019; Fellner and Lederer 2020).

#### Space

The concept of space is changing in different research domains (Gotham 2003; Bourdieu 2018). Since the object of this paper is the material cycles in the built environment, we consider the physical rather than the social characteristics of space from a geographic perspective. For instance, the effects of building/network density and land use on the resource cycles in the built environment have been mentioned sporadically (Stephan and Athanassiadis 2017; Heeren and Hellweg 2019; Bogoviku and Waldmann 2021; Augiseau and Kim 2021a). We, therefore, limit the scope of space to the characteristics of the spatial distribution patterns in the built environment within/between locations and regions, including the conventional spatially quantified and spatially dynamic characteristics such as density, dynamics, supply and demand, and distance. It is essential to point out that locations and regions are two fundamental concepts of geographic space. Geographic location can be represented in the absolute terms of the coordinate on a grid of two to four dimensions, and region refers to an area with one or more characteristics that distinguish it from the surrounding areas.

### Research framework.

**Fig. 1:**
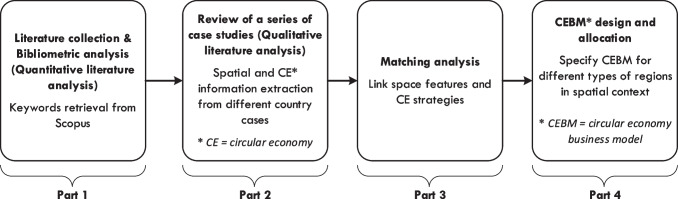
The research framework based on the literature review

A literature review is an integral part of any research field. Assessment and analysis of relevant literature through literature review can summarize research clues and identify possible research gaps, which will help strengthen the research area (Kamble et al. 2018). We reviewed and analyzed relevant studies to understand the relationship between space and the CE in the built environment for this study. The review framework of the paper is shown in Fig. 1. The general trends in the field were first identified from bibliometrics through literature search and analysis (step 1). Then, country/region-specific cases were selected from the acquired literature to extract information about space and CE further and analyze the linkages (step 2 and step 3). Finally, in step 4, the selection of CE strategies and the design of CEBM in a spatial context is performed based on the results of steps 1–3.

### Retrieval strategy

The primary method underpinning the study is a systematic review of the literature, which involves a bibliometric analysis, critically assessing the literature systematically, and synthesizing the findings into a coherent statement. Our retrieval from the Scopus database covered all the core terms of this paper, i.e., publications related to space, CE, built environment, and materiality, and then filtered the spatial features and their impacts on CE strategies from the retrieved results. We obtained peer-reviewed publications from scientific journals and conferences in English from 2000 to 2021. Three types of research were considered: (1) case studies discussing the material flows and stocks in the built environment; (2) case studies discussing CE in the built environment; and (3) review and descriptive studies that mentioned case studies focusing on dynamics and circularity of building materials.

To diversify the results of this systematic review, we adopted words with similar meanings to replace the four keywords in the publication retrieval, which were gathered from relevant literature review and concept description papers (Korhonen et al. 2018; Morseletto 2020; Gallego-Schmid et al. 2020). The operator “AND” concatenate the keywords in the four research areas in Table 1. Then, “TITLE-ABS-KEY” is used to limit the search scope to titles, abstracts, and keywords. “PUBYEAR > 1999 AND PUBYEAR < 2022” and “LIMIT-TO (LANGUAGE, “English”)” were served to narrow the scope of retrieval to publications from 2000 to 2021 and the English language.

**Table 1:** Retrieve keywords and strings

Research areas	Keywords	Boolean operators (among keywords)
Built environment	built environment, construction, building, infrastructure, urban, rural, city, house, residential, home, domestic building, commercial building	OR
Materiality	material consumption, material efficiency, urban metabolism, material flow, material stock, material dynamic, in-use	OR
Circular economy	circular economy, circularity, reverse logistic, recycle, reuse, recovery, closed-loop, recycling, waste management, anthropogenic material, secondary material, urban mining, durability	OR
Space	space, spatial, spatiality, geography, region, territory, place, location, distance, density, spatiotemporal	OR

### Cases analysis

Not all retrieval results provide a comprehensive and direct description of space; therefore, we strategically select representative case studies without including review papers for thorough analysis of different spatial characteristics. These cases should encompass a broad geographical range, including subjects from various continents. Urbanization, as the central physical expression of the built environment and the primary space occupation of human activity, is deemed the main driver of resource utilization in the built environment, particularly in cities (Yeh and Huang 2012; Næss 2016). Major construction material flows and stocks are concentrated in cities, covering only 2% of the earth’s surface but consuming 75% of its resources (Madlener and Sunak 2011; Yu et al. 2018). Furthermore, urbanization as a crucial spatial feature can serve as an intermediate element to connect other spatial indicators. Our strategy is to classify the selected case areas according to their urbanization level and analyze how specific spatial characteristics play a role in influencing material circularity in these areas. A standard measure of urbanization level is the urban population share, which describes the percentage of the population living in urban areas. This method has also been adopted by the World Bank and the CIA World Factbook (US CIA 2022). We take this measurement here and use it to classify selected cases. Therefore, in the case study section, we aim to choose cases distributed across different continents with differentiated levels of urbanization, and analyzing these representative cases can reveal information that is not visible in the bibliometrics.

## Results

### Literature panorama and structure

**Fig. 2:**
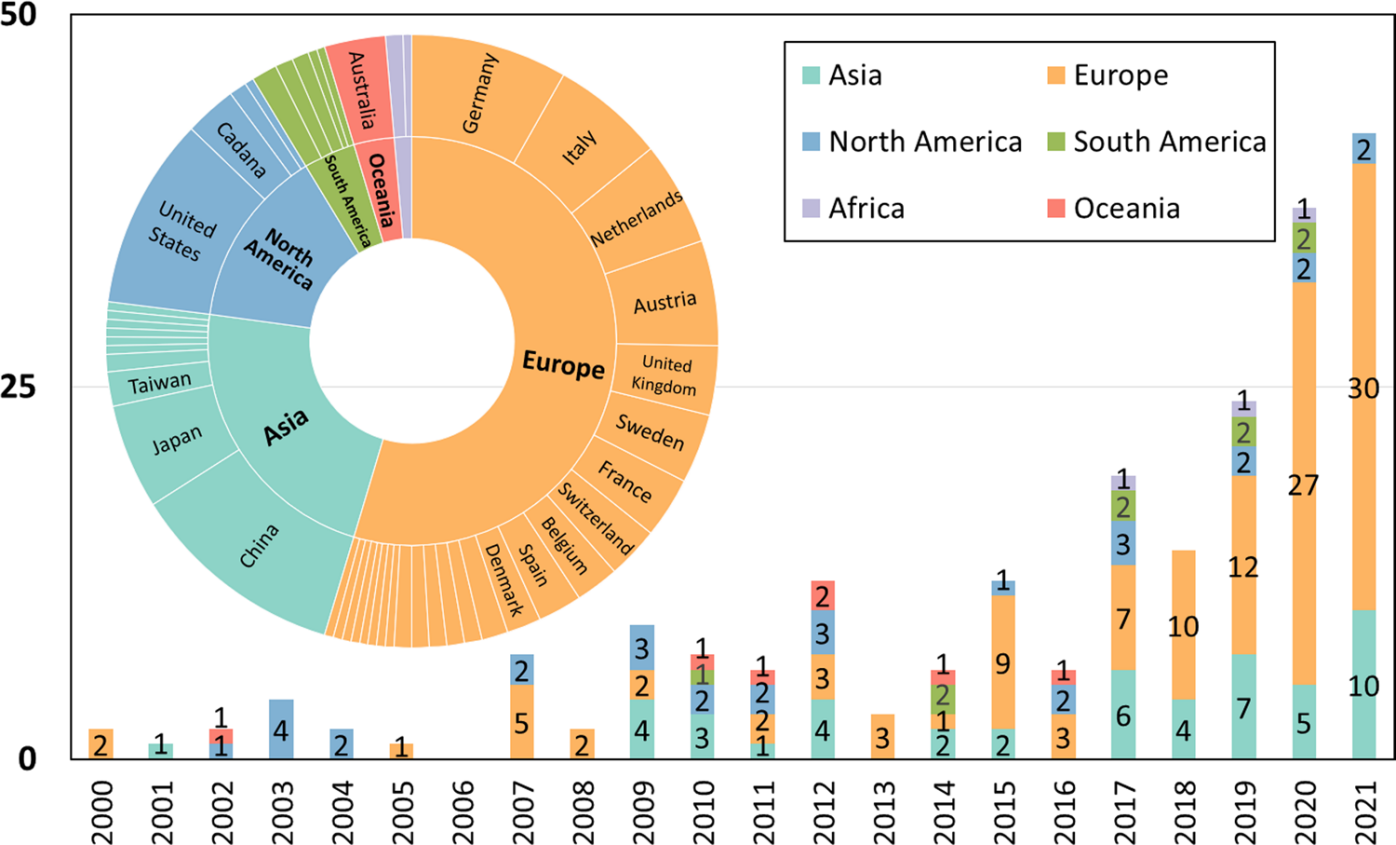
Number and geographic distribution of the retrieved publications of this review by year and by country or region

The 218 publications reviewed in this studies demonstrate a growing interest in the circularity of materials in the built environment, particularly in relation to space. As shown in Fig. 2, research in this field began early but has seen a significant increase in recent years, with the majority of studies dating from 2017 to 2021, and the earliest publication reviewed from 2000.

Geographically, more than half of the first authors’ affiliations in the reviewed publications are derived from European countries, led by Germany (18), Italy (13), and the Netherlands (12). The following sources are Asian countries, including mainland China (25), Japan (12), and Taiwan (4). The composition of research sources from the remaining four continents is more straightforward—than the composition of the research sources in the remaining four continents. In short, their most significant contributors include the USA (22) and Canada (6) in North America, Brazil (3) in South America, Australia (7) in Oceania, and South Africa (2) in Africa.

**Fig. 3:**
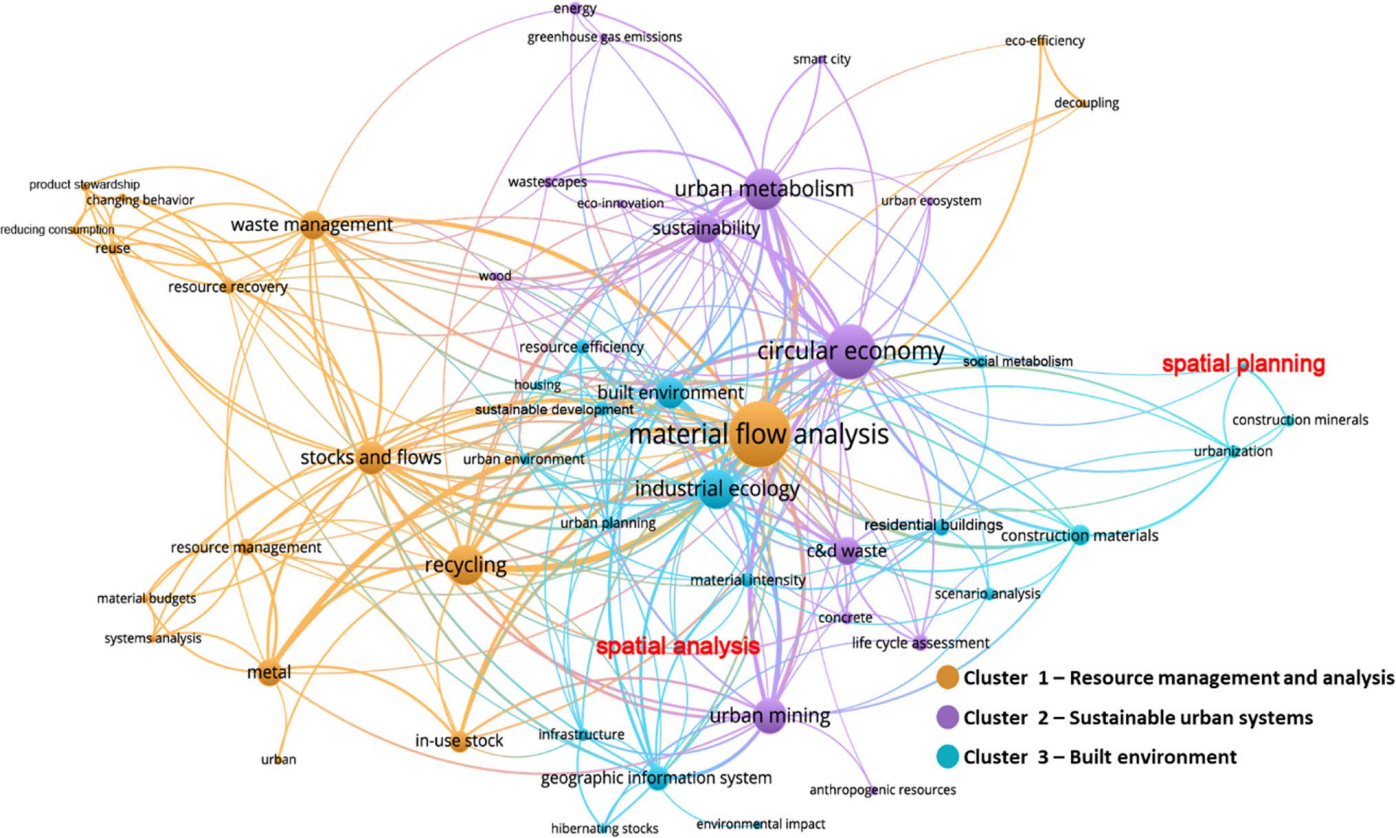
The map of author keywords co-occurrence in the selected publications

Term co-occurrence is a part of the bibliometric method used to detect main areas of interest and to identify topics/sub-topics that co-occur more frequently as research clusters (Sharifi 2021). These clusters are identified and analyzed by software based on the strength of connections between terms. In other words, terms that co-occur frequently form a cluster. In this paper, author keywords were selected as the terms to be analyzed. After setting a minimum number of keywords occurrences, merging the words with the same meaning (e.g., “LCA” and “life cycle assessment”), and removing terms that are not relevant (e.g., “method,” “study,” and “analysis”), the measurement of the co-occurrences has made it possible to derive the following evidence graphically described in Fig. 3. Three clusters were identified by grouping closely related keywords, and the links between the keywords highlight the relevance of the research hotspots. Straightforwardly, the 3 clusters focus on *resource management and analysis*, *sustainable urban systems*, and *built environment*, respectively. The most frequent keywords (the size of the circle of an item is determined by the weight of the item) are “material flow analysis,” “circular economy,” and “industrial ecology,” which represent the research object (construction materials), research aim (CE), and research method (industrial ecology). Lines link the relationships between keywords. The closer two keywords are to each other and the thicker the connecting line, the stronger their relevance in terms of co-citation links. These expressions help us find spatially relevant information in this figure, albeit relatively little. We found two keywords belonging to cluster 3, namely, “spatial planning” and “spatial analysis,” which showed weak associations with other keywords in the cluster. We can obtain the following superficial space-based evidence from the analysis of keywords: (1) Mineral materials are a category that receives special attention in the resource cycle; (2) spatial information is linked to the dynamics of urban building materials; (3) MFA, industrial ecology, and GIS serve the spatial analysis in this field. The specific relationship between space, materials, and CE still requires further analysis of the publications.

### Distribution of case studies

**Fig. 4:**
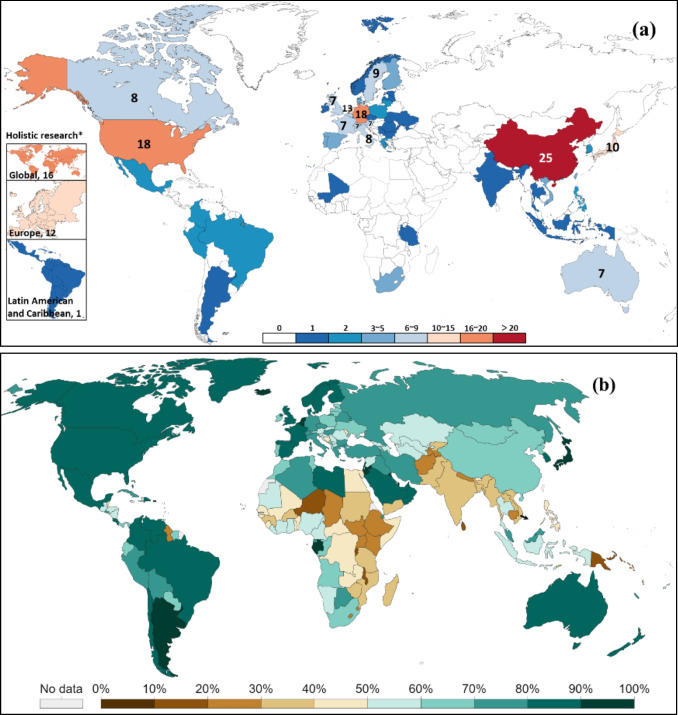
The geographical distribution of **a** research scope in publications between 2000 and 2021; **b** urbanization level by population in 2020 (World Bank 2021). Note: Publications that do not contain case studies have been excluded. *Holistic research means research conducted from a macro perspective, generally continental and global

The 218 publications contain lots of research using actual regions as cases. The case studies not only cover six continents (Asia, Africa, North America, South America, Europe, and Oceania) but also contain the regions with different urbanization levels. The geographical distribution of the case studies is shown in Fig. 4, with a large concentration in Europe, North America, and East Asia, and contains 28 cases conducted from a global and Europe-wide perspective. It can be found that most studies are concentrated in the global north or highly urbanized and developed countries. There are no targeted case studies in many countries in Africa and West Asia, which are also regions with low levels of urbanization. But the United Nations and World Bank estimate that Asia and Africa contribute the majority of the world’s labor-intensive industries and that global population growth and urbanization trends over the next 50 years will be dominated by Asia and Africa (UN DESA 2019; UNIDO 2019; Wahba et al. 2021). The current research gaps in this region need to be filled in the near future.

### Analysis of cases

**Fig. 5:**
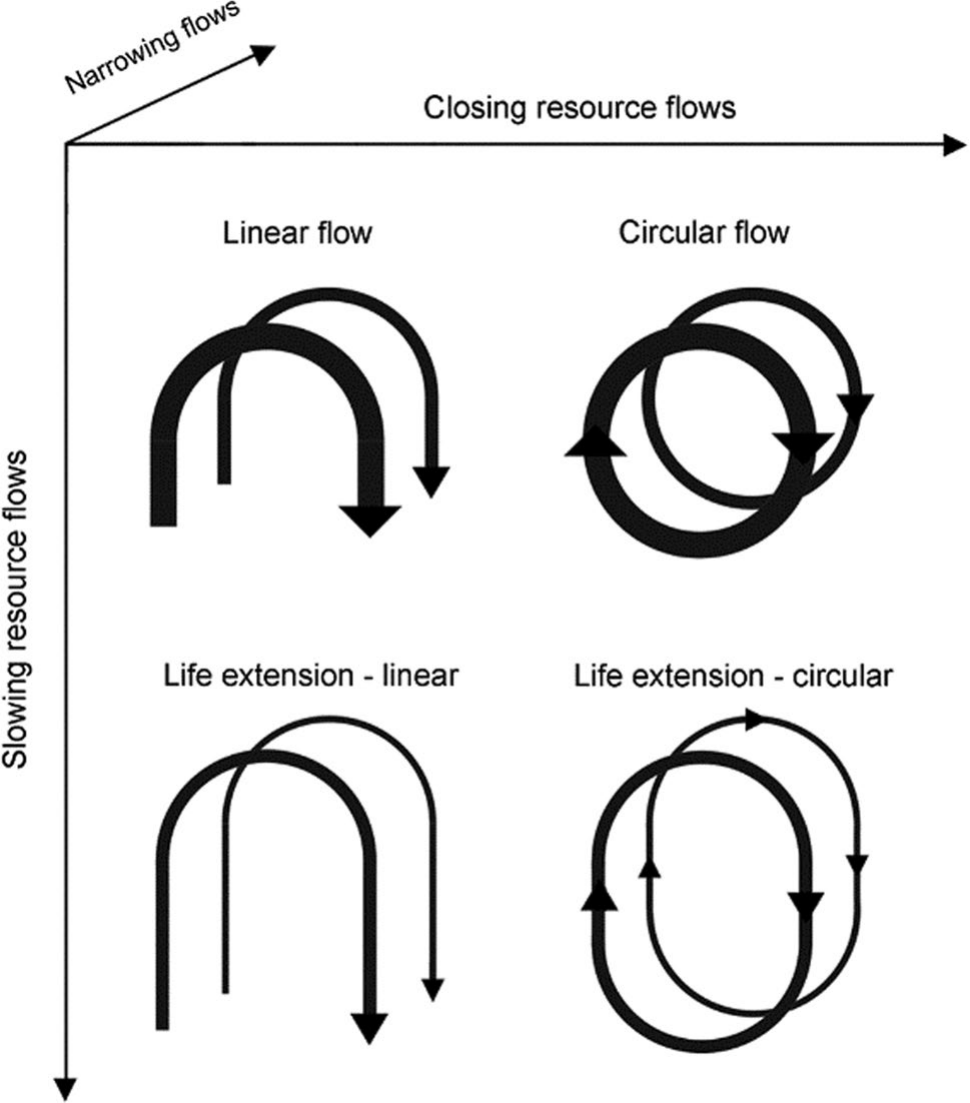
Categorization of three main CE strategies (Bocken et al. 2016; Gallego-Schmid et al. 2020)

After conducting a thorough review of the cases, we have gathered evidence on the material cycles and assigned them into three stragies: narrowing, closing, and slowing. Narrowing involves enhancing resource efficiency and avoiding unnecessary consumption, which a can be applied to both the linear economy and CE models. Slowing entails prolonging the service life of products by resource flow control. Closing refers to creating a closed loop between post-use and production, enabling a circular flow of resources, as illustrated in Fig. 5 (Bocken et al. 2016).

The cases are selected based on the principle of covering as wide a geographical area of the globe as possible. We summarized CE clues found from different countries in Tables 2, 3 and 4.

**Table 2:** Impact of space features on narrowing resource loops

Spatial features		Impact on CE	Region	Reference
Availability of materials	Availability of natural materials	Continued available and low-cost local materials can reduce material efficiency in the built environment	Tanzania	Colley et al. (2021); Moses and Mosha, (2020)
Density	Population density	Material utilization efficiency is proportional to population density	France; Brazil	Augiseau and Kim, (2021b); Condeixa et al. (2017)
	Building density	There is an inverse relationship between the stocks of construction materials in buildings and traffic infrastructure	Germany; China	Mao et al. (2020); Schiller (2007)
Terrain	Coastline and elevation	Construction materials are used more efficiently in coastal areas and lower elevations on islands	Grenada	Symmes et al. (2020)
	Hills and elevation	Materials are more efficiently utilized in areas with gentle terrain because the same structure does not require additional materials for safety in plain areas	China	Mao et al. (2020)

**Table 3:** Impact of space features on slowing resource loops

Spatial features		Impact on CE	Region	Reference
Availability of materials	Availability of anthropogenic materials	The insufficient quantity of components resulting from building demolition and the not dimensional coordination could hinder the combination of components from distinct buildings into the construction of new buildings	Brazil	da Rocha and Sattler (2009)
		Making it clear what is available after demolition can help overcome barriers to the reuse of building structural materials	The UK	Densley Tingley et al. (2017)
Availability of land resources	Availability of construction land plot	The reduction in available construction land plots can shift construction activity to refurbishing existing settlements to increase durability and avoid new build	Germany	Keßler and Knappe (2013)
Demand for materials	Demand for anthropogenic materials	The lack of a demanding market is an obstacle to the reuse of building structural materials	The UK	Densley Tingley et al. (2017)
Density	Population density	The higher the population density, the lower the annual building refurbishment rate (as a percentage of building floor area)	France	Augiseau and Kim (2021b)
	Building density	Due to site space constraints, construction sites for dense buildings usually do not have on-site classification for direct reuse	China	Bao et al. (2020); Jin et al. (2017)
	Urban density	In the regions with low-density urban development, materials are prioritized for infrastructure maintenance rather than new build	Germany	Schiller (2007)
Distance	Transportation distance of waste materials	The disparity between the location of stocks of reclaimed items and added costs due to transport and storage are barriers to the reuse of building materials	Norway; Brazil	Condeixa et al. (2017); Knoth et al. (2022)
Urbanization	Urbanization level	As urbanization increases, there is a higher probability that construction activities serve the repair and retrofitting of constructions. Conversely, urbanization is lower, and construction activities are more likely to serve as demolition and new build	The USA; Vietnam; EU-25	Miatto et al. (2017); Nguyen et al. (2019); Wiedenhofer et al. (2015)
	Urbanization dynamics	The urbanization dynamics affects the durability of building materials. Rapid urban expansion can lead to short-lived buildings	China	Cao et al. (2019); Huang et al. (2018)
Terrain	Coastline and elevation	Construction materials are more exposed to extreme weather and climate change in islands’ coastal and low-elevation areas, which shorten their service life	Grenada	Symmes et al. (2020)

**Table 4:** Impact of space features on closing resource loops

Spatial features		Impact on CE	Region	Reference
Availability of materials	Availability of natural materials	The shortage of natural construction materials could facilitate the introduction of recycled materials made from C&D waste into the material cycle (and vice versa)	Vietnam; Grenada	Hoang et al. (2021); Lockrey et al. (2016); Symmes et al. (2020)
		The availability of non-metallic mineral raw materials affects the use of secondary materials when there is a shortage of supply; the substitution with secondary materials increases (and vice versa)	Germany	Schiller et al. (2017a)
		Recycling activities are concentrated in areas where there is insufficient local natural aggregates	The USA	Robinson (2004); Robinson et al. (2004)
		Sufficient natural resource reserves in the surrounding area provide direct imports and impede urban mining, although plenty of anthropogenic material is flowing out	France	Augiseau and Kim (2021a)
	Availability of anthropogenic materials	(1) Even with increased recycling rates, the secondary materials available in large cities would only marginally replace primary materials. In Vienna, improving the recycling rate would only reduce the use of primary resources by 7% (2) Sufficient anthropogenic materials do not necessarily promote material recyclability. Under technical limitations, secondary materials are applied to a minimal extent, and large amounts of low-quality recycled minerals have to be exported after satisfying local demand	Austria	Lederer et al. (2020)
Availability of land resources	Availability of dumping sites	The higher the available capacity of the landfill, the fewer possibilities there are for the material to be recycled	Germany; the UK	Casas-Arredondo et al. (2018); Keßler and Knappe (2013)
		Adequate and weakly regulated landfills around the city reduce the material circularity	Vietnam	Hoang et al. (2020); Lockrey et al. (2016)
Demand for materials	Demand for natural materials	Increased demand for materials can promote the use of secondary resources	China	Tang et al. (2021)
	Demand for anthropogenic materials	There is a higher capacity to process and produce recycled aggregates in areas with high demand for concrete	Germany	Tsydenova et al. (2021)
		Low demand for secondary materials can hinder the circularity of materials	Japan	Hashimoto et al. (2007)
Density	Density of population	Aggregates recycling activities are located in areas with high population density	The USA	Robinson (2004); Robinson et al. (2004)
	The density of construction and demolition activities	Most of the C&D waste recycling activities are located in areas with intensive construction and demolition activities	Germany; China	Abdelshafy and Walther (2021); Song (2015); Tsydenova et al. (2021)
	Building density	High-density built-up areas make it difficult to conduct recycling activities and are less likely to use recycled materials on-site	China	Bao et al. (2020); Jin et al. (2017)
	Road density	The use of secondary and recycled aggregates is higher in areas with dense road networks	The USA	(Robinson, (2004); Robinson et al., (2004)
Distance	Transportation distance of waste materials	The transport distance of the waste material determines the method by which the waste is processed. Concrete debris with low added value is mainly transported to the outskirts for simple recycling to produce low-quality aggregates	Vietnam; Kuwait	Kartam et al. (2004); Lockrey et al. (2016)
	The radius of the urban area	There are more recycling activities in the suburbs with the closest radius to the downtown area	China	Chen and Liu (2021)
	Distance between recycling sites and major road networks	Concrete aggregates recycling activities are located close to major road networks	The USA	Robinson (2004); Robinson et al. (2004)
	Transportation distance of recycled materials	The profitability and utilization of recycled materials are higher when artificial quarries are closer to the construction site or product manufacturing site than traditional quarries	Europe; Germany	Gálvez-Martos et al. (2018); Keßler and Knappe (2013)

#### Narrowing loops

Spatial factors can affect the efficiency and utilization of materials. The concept of “narrowing,” which aims to improve material efficiency by making consumption less or denser, has limited research. Technical means are currently considered the primary driver of material efficiency and reduction, with space considerations playing a secondary role as either an incentive or obstacle. The reviewed articles focus on evaluating material usage, as shown in Table 2, with cases from five continents addressing material availability, density, and local terrain.

In Tanzania, the cost-efficient housing issue has become more challenging because population growth is not proportional to people’s income (Andreasen and Møller-Jensen 2017). Soil and sisal were used extensively in the country’s construction to make reinforced adobe (unkiln-fired dried mud brick) instead of concrete and bricks that are locally and readily available and could be used for basic construction activities after simple processing. Even though the operation controls the cost of materials, it fails to reduce the intensity of materials or even increases consumption of traditional building materials as a substitute for industrial materials (Moses and Mosha 2020).

Findings from Europe, China, and Brazil suggest that density affects material efficiency. Augiseau and Kim (2021a) compared construction material dynamics in four French regions and found a significant negative correlation between per capita material consumption and population density. The population density of Paris is 4 times that of Petite Couronne and 9 times that of Grande Couronne, but the per capita inflow of building materials is only 1/3 that of Petite Couronne and 1/5 that of Grande Couronne (Augiseau and Kim 2021a). The case of Brazil on material consumption provides similar results. In Rio de Janeiro, areas with greater population density have a smaller material mass per unit of floor area (Condeixa et al. 2017).

When material stock density is used to represent material use efficiency, the role of spatial characteristics is reflected in the case of several countries. In particular, there is a high probability that material efficiency in transportation networks is determined by building density (Mao et al. 2020). The analysis of Schiller (2007) implies that material efficiency in infrastructure in German construction decreases with rising built-up density. The other factor is the terrain. By estimating the stock distribution of construction materials in Grenada, it is evident that material stocks in constructions are not only denser in the coastal and low elevation areas of this small island country but also highly vulnerable to sudden loss of stock due to extreme weather and climate (Symmes et al. 2020). In Beijing, Mao et al. (2020) used GIS to map and characterize the material stock of the entire city. The material distribution pattern in Beijing can be easily summarized from their results. Materials are more concentrated in southeastern Beijing because the southeast is a gently sloping plain toward the Bohai Sea that easily connects to other large cities, while mountains surround the western and northern parts of Beijing.

#### Slowing loops

“Slowing” refers to product life extension through repair, maintenance, remanufacturing, and reuse. The emphasis of slowing loops in building materials is on the construction components and structures. In the collected publications, most researchers prioritized the building’s lifespan, reuse of materials, and maintenance of the structures.

As shown in Table 3, material availability, demand for recycled materials, building density, and transportation distance of waste were found to influence the reuse of materials. da Rocha and Sattler (2009) discussed that the key to holistic reuse is the quantity of structures, but the premise of reuse is dimensional coordination which affects the combination of components between new buildings and reused products. The reuse of waste components or materials generated by construction activities needs to be considered in the context of transportation economics. For instance, demand being located beyond the transportation range has been identified as a barrier to reuse in Norway, Brazil, and the UK studies. Building density can have an impact on reuse by controlling the available space on the construction site. Jin et al. (2017) and Bao et al. (2020) state that the reuse of building materials in Hong Kong is dependent on on-site classification, but it is not easy to find sufficient spaces to achieve on-site sorting in a high building density environment.

Population density, urban density, and urbanization levels affect the maintenance, repair, and retrofitting of existing structures. Augiseau and Kim (2021b) analyzed refurbishment rates of buildings in three regions of France (Paris, Petite Couronne, and Grande Couronne). They found that refurbishment rates were lower in areas with higher population densities. The negative correlation with urban density is pronounced for the frequency of infrastructure maintenance. Infrastructure maintenance is a higher priority than new builds in low-density urban scenarios. Likewise, urbanization is an important characteristic, and areas with high urbanization levels tend to stabilize the built environment, preferring to use maintenance and retrofitting rather than rebuilding of built-up areas.

We also summarized the impact of land resource availability, urbanization, and terrain on slowing. For instance, in Germany, building activities are constrained by limited land resources, which will lead to a greater focus on preserving and restoring existing buildings in the core areas of settlements (Keßler and Knappe 2013). The impact of urbanization dynamics on building activity is fundamental. China has just undergone high-speed urbanization, but the process has brought widespread short-lived buildings to several Chinese cities. Terrain plays a role in the slowing system as an easily overlooked spatial feature. Symmes et al. (2020) described buildings on small islands. The more they are located in coastal and low elevation areas, the more likely they are to be affected by sea-level rise and seawater erosion, thereby reducing the durability of materials and structures.

#### Closing loops

Recycling is the primary method of creating a closed loop of resources. The impact on the “closing” strategy on spatial characteristics is discussed in Table 4. Studies on the material availability have primarily centered on non-metallic mineral materials, which make up the majority of construction materials. The availability of natural mineral materials discourages the use of recycled materials and acts as a disincentive to recycling and urban mining activities. However, the impact caused by the availability of anthropogenic materials would be far weaker. A study by Lederer et al. (2020) noted that Vienna generates 1.8 million tonnes of C&D waste per year, of which 1.7 million tonnes are recycled, but only 400,000 tonnes of the main raw materials can be replaced. Targeted recycling and self-sufficiency are challenging to achieve in Vienna. Even though the recycling rate is high, recycled materials with poor quality are challenging to adopt widely. They have to be exported in large quantities due to the limitations of recycling technology. An implicit factor related to material availability is the demand for building materials. High demand and insufficient supply of natural resources is a scenario for promoting recycling activities (Robinson et al. 2004; Hashimoto et al. 2007; Tsydenova et al. 2021). While recycling is a key method, landfilling is also a significant outflow. They can consume valuable land resources, and in areas with strict regulations, the capacity of legal landfills directly affects the rate of recycling due to their lower disposal costs compared to recycling (Keßler and Knappe 2013; Casas-Arredondo et al. 2018). There are exceptions. However, the lack of strict regulation and the sufficient number of illegal landfills—as is reported for the case of a city in Vietnam—also reduce the possibility of material recycling (Lockrey et al. 2016; Hoang et al. 2020).

Population density, building and infrastructure density, and construction activity density are used to determine overall density. Building density plays a role in promoting reuse and on-site recycling, as limited space in high-building-density areas like Hong Kong can make on-site recycling challenging. A study by Robinson et al. (2004) in Virginia and Maryland found that areas with a population density of over 30 people/km^2^ were 2.3 to 2.4 times more likely to have both types of recycling sites for concrete and asphalt. Additionally, recycled aggregates were more likely to be used and supplied in areas with dense road networks due to easier processing for recycled aggregates applied to road projects than aggregates for buildings.

Another understanding of high density is to reduce the distance waste materials are transported. It is financially feasible to set up recycling facilities and use recycled materials directly in areas with frequent construction and demolition activities or close to roads (Song 2015; Tsydenova et al. 2021). Indeed, this is the case in Virginia and Maryland, where a majority of pavement aggregate recycling sites are located in proximity to major roadways and in densely populated suburban areas. Specifically, 64% of these sites are situated within 4.8 km of an interstate, and 82% are located in areas with a population density of over 400 people/km^2^ (Robinson et al. 2004). Beyond the density, the transport distance of recycled material is also essential. Studies across Europe have shown that the profit margin on recycled aggregates depends on the localization of the resource, which has to be closer than conventional quarries in order to overcome the barriers proposed in the EU Waste Framework Directive related to (i) availability, (ii) economics, and (iii) acceptability of waste recycling (European Parliament and Council of the European Union 2008; Gálvez-Martos et al. 2018).

### Matching analysis

These discovered spatial features are not independent. In order to correlate multiple spatial dimensions with CE strategies, we first need to find out how the spatial features are related to each other. Table 5 shows all the spatial characteristics summarized from the literature and the causal relationships. “Positive” represents positive correlation, i.e., a positive change in factor “A” causes a positive change in factor “B.” Conversely, “negative” means negative correlation, i.e., a positive change in factor “A” causes a negative change in factor “B.” Positive changes specifically refer to the increases in the value of spatial characteristics, such as higher density and longer distance. A correlation that needs to be emphasized is the “negative” between the urbanization level and the urbanization dynamics. By counting the relationship between the urbanization level (2020) and the urbanization growth rate (2015–2020) for 186 countries/regions, a negative correlation between them was obtained (see Fig. 7). It indicates that the higher the urbanization level, the slower their urbanization process.

**Table 5:** Correlations between spatial features

Spatial features		Correlation	Description	Reference
Terrain*	Distance	Negative	Rough terrain conditions can increase spatial distances	Burdett et al. (2015)
	Density	Positive	Favorable terrain, including low elevation and flat terrain, can increase the density of settlements, population, buildings, etc	Mubareka et al. (2008)
	Availability of materials	Positive	Rough terrain conditions hinder the availability of materials and increase logistical challenges	McDougall and Williamson (2014); Sharma and Bansal (2018)
Density	Distance	Negative	The distance decreases as the density increases	Guan (2019); Resch et al. (2016)
	Availability of land resources	Negative	Increased density reduces the availability of land resources	Hasse and Lathrop (2003)
	Urbanization level	Positive	Urbanization is the result of increased density	Gaughan et al. (2016); Polinesi et al. (2020)
Distance	Availability of materials	Negative	Short distances increase material availability	Anastasiou et al. (2015); Coelho and de Brito (2013)
Urbanization level	Distance	Negative	High urbanization level shortens spatial distances	Li et al. (2013)
	Availability of land resources	Negative	Land availability is lower in highly urbanized areas	Qian et al. (2015); Verhoeve et al. (2015)
	Urbanization dynamics	Negative	The rate of urbanization decreases as the level of urbanization increases	Data analysis
Urbanization dynamics	Demand for materials	Positive	The growth of urbanization increases the demand for materials	Fry (2011); Yeh and Huang (2012)
Availability of land resources	Demand of materials	Positive	The available land resources for construction activities increase the demand for materials	Novellino et al. (2021)
Demand of materials	-			
Availability of materials	-			

*Positive changes in terrain refer to the flattening of the topography, the decrease in elevation, the reduction of hills, etc

**Fig. 6:**
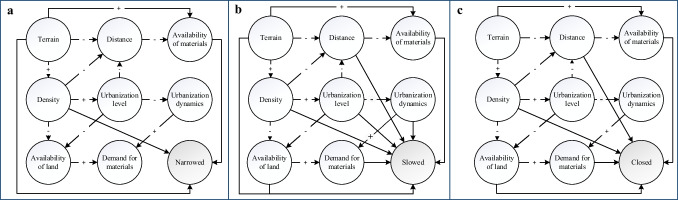
Relationships between spatial features and CE strategies (**a** narrowed; **b** slowed; **c** closed)

These relationships are visualized according to the explicit associations in Tables 2–5. Each link is assigned a polarity, either “ + ” or “–.” The signs in Fig. 6 indicate the positive and negative associations between the spatial dimensions in Table 5. A positive sign means the variable increases or decreases in line with the arrow variable, whereas the negative sign indicates that the variable goes in the opposite direction of the arrows. The arrow line between space and CE strategy indicates that a spatial factor can influence a specific type of CE pattern. It can be seen that slowing is the most sensitive CE strategy and is influenced by all retrieved spatial features. While narrowing is relatively less affected, only density, material availability, and terrain can clearly place the influence.

**Fig. 7:**
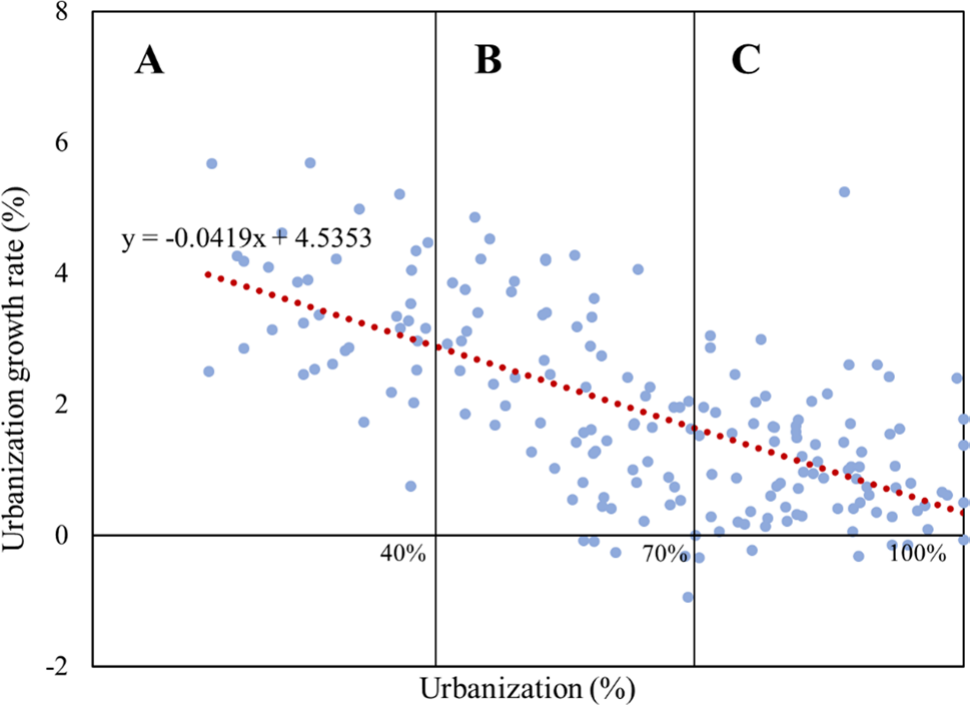
Correlation between urbanization level and urbanization dynamics. Note: including 186 countries/regions; **A** low urbanization level, **B** middle urbanization level, and **C** high urbanization level. The very initial stage of urbanization is not considered because it contains only a very few countries

To develop appropriate CEBM for regions with varying spatial characteristics, this study explores other spatial features by analyzing correlation between the obtained spaces, beginning with urbanization level as it is easily quantifiable and closely related to construction activities. The urbanization levels (0–100%) can be roughly divided into three categories from low to high, which are A (low, 0–40%), B (middle, 40–70%), and C (high, 70–100%) (see Fig. 7).

**Fig. 8:**
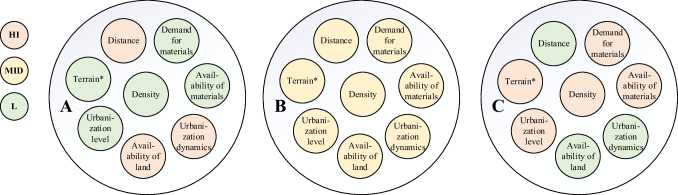
Classification of regions based on the relationships between spatial features. Note: *High in the description of the terrain represents flat and low elevation terrain favorable for construction; low represents dense hills and high elevation terrain unfavorable for construction activities, and medium is in between

By defining the urbanization level, we can infer the spatial features through correlation. For instance, when disregarding the exceptions in Fig. 7, regions with high urbanization levels have low urbanization dynamics (inverse proportion) and high densities (direct proportion). Thus, classifying areas by urbanization level can provide a more detailed spatial description and can be grouped into three types, as shown in Figs. 7–8.

### CEBM design and allocation

While organizing the spatial information, potential CEBMs based on circular resource strategies were summarized (see Table 6). The table’s framework is taken from Lüdeke‐Freund et al. (2019), from which it is clear that the CEBM patterns are mainly based on the design of slowing and closing loops, while few models serve to narrow the material loops. Table 6 was extended by combining the clues from Fig. 6. In terms of their impact on resource cycling strategies, material availability is the most crucial spatial characteristic to consider, followed by terrain, land resource availability, material demand, density, and distance. Urbanization should be considered last when assessing the importance of spatial characteristics.

**Table 6:** Major circular economy business models (CEBM) patterns

Spatial features affecting the material cycle	Resource cycling strategy	Major CEBM patterns	CEBMs supporting the patterns
Terrain; density; availability of materials	Narrowing	Reduction	Resource efficiency; less material-intensive; product lightweighting
Terrain; availability of land resources; density; urbanization level; urbanization dynamics; distance; availability of materials; demand of materials	Slowing	Repair and maintenance	Repair; product life extension; classic long-life model
		Reuse and redistribution	Reuse/refurbish/maintain/redistribute/next-life sales; reuse; product life extension
		Refurbishment and remanufacturing	Remanufacturing/next-life sales; upgrading; product life extension; extending product value
Availability of land resources; density; distance; availability of materials	Closing	Recycling	Closed-loop production; dematerialization; recycling and waste management
		Cascading and repurposing	Multiple cash flows/multiple revenues; co-product generation from waste
		Organic feedstock	Co-product generation from waste; circular supplies; resource recovery; industrial symbiosis

Tracking these relationships enables us to not only classify regions based on various spatial characteristics but also to design appropriate CEBMs. However, some CEMBs in Table 6 are not suitable for the built environment, particularly “organic feedstock.” Therefore, Table 7 also provides specific resource cycling strategies and refines the CEMBs to align with the built environment.

**Table 7:** CEBM allocation and design according to regional characteristics

Region type by urbanization level	Regional characteristics	Major construction activities	Primary applicable resource cycling management strategies	CEBM
A – Low	Regions developing at a high rate are using sufficient material stocks for new construction-based urban construction	Construction	Narrowing	Resource efficiency; less material-intensive; product lightweighting
B – Middle	Between A and C. Regions in this category have both renovation, demolition, and construction activities	Demolition, construction, and renovation and decoration	Closing	Closed-loop production; recycling and waste management; co-product generation from waste; circular supplies; resource recovery
C – High	The urban construction of such regions has been completed. In this stage of development, regions do not involve significant demolition and construction activities but rather renovation and maintenance	Renovation, retrofitting, and repair	Slowing	Repair; life extension; reuse/refurbish/maintain/redistribute; reuse; upgrading

This study defines regions belonging to category *C* as areas where urban construction has been completed and the built environment has stabilized. However, during the process of urbanization and extensive construction, the availability of materials in the surrounding area has decreased due to high consumption. At present, there are no major demolition and new build activities in the region, with only retrofitting, renovation and repair, resulting in low demand for materials. In renovation activities, the removed building components are easily reused unlike the bulk material from demolition activities that can only be recycled or landfilled. Thus, the strategy of slowing is proposed to be adopted primarily in such areas. For instance, refurbishment and remanufacturing can directly reduce the landfilled flow to ease the shortage of land and cope with the shortage of raw materials. Furthermore, the technical updating of old components for material reductions during maintenance and repair has been proven in the case of slowing, and it can be combined with narrowing strategies.

Regions in category *A* are characterized by rapid development and are experiencing large-scale urban expansions and frequent new build activities, which consume large reserves of materials. This phase is the reasonable period to consider narrowing strategies in the built environment, as the reduction of material consumption and the improvement of material efficiency in the construction activities of new buildings are most evident (Gallego-Schmid et al. 2020). Secondly, the positive correlation between the urbanization level and the economic development level is relatively well established (Li and Ma 2014), suggesting that the financial situation in these areas with low levels of urbanization is potentially poor as well. But CEBMs based on slowing and closing strategies are highly diverse. They involve various actors which place high demands on technology, services, and organizational scheduling, thereby making those inapplicable to this type of region with a low economic level (Tong et al. 2018). The economic benefits of recycling construction materials are still controversial, so it is difficult to accept these models if the economic benefits of recycling cannot be guaranteed in areas with sufficient materials.

Regions in category *B* fall in between A and C, having a max of retrofitting and C&D activities. Slowing and narrowing strategies are challenging to implement for circular management of waste bulk building materials from demolition activities; therefore, recycling is a viable alternative to prevent these materials from ending up in landfills. In shaping the built environment in this region, accessible materials are being depleted, but with the reduced construction activities, it is advisable to gradually adopt closing strategy models in areas of low density to save remaining materials and in areas of sufficient land resources and economic resources, early deployment of recycling facilities in beneficial for CE transition and avoiding land occupation.

## Discussion

### Examples of CEBMs used in A, B, and C regions

#### “Narrowing” strategy in low-level urbanization region

As defined in this paper, low urbanization levels are countries/regions with a growth rate of urbanization of less than 40%. Many of these countries are located in Africa and South Asia (see Fig. 4b). Tanzania, located in Africa, has an urbanization level of 35.2% in 2020 and an urban population that is growing at a rate of 5.22%/year. However, due to the high initial investment and long payback period, few facilities are closing and slowing the material loops in Tanzania (Mosha 2017; Todd et al. 2019). So locally adapted narrowing solutions have been developed for processing and use of building materials to reduce material intensity. The growing urban population and expanding urban areas have increased the demand for building materials. One of the prerequisites of the urbanization trend for this low-income region is to control the cost of materials. We discussed in "Narrowing loops" that using entirely cheap materials and rough processing can increase consumption and fail to achieve narrowed. But reducing the percentage of these materials used can make a difference and become a consideration for modern construction in Tanzania. In some urban areas of Tanzania, a program of mixing a certain percentage of industrial materials with local cheap and available natural materials to make products has been applied. For instance, the bricks used to build the walls are made from a mixture of cement and soil (soil–cement interlocking bricks), and the tiles used to create the roof are made from sand, cement, and sisal fiber (sisal concrete roofing tiles). Both of these typically indigenous materials are produced by pressing machines rather than kiln-fired, allowing different sizes and types of bricks with good matching and low waste rates to be made without cutting. Construction cost and concrete consumption of soil–cement interlocking brick wall and roofed with sisal concrete roof tiles are significantly reduced by 40% from that of the same structure but of sand-cement block wall and roofed with corrugated iron sheets (Moses and Mosha 2020). The example from Tanzania illustrates that the use of technologies and operations based on narrowing strategies in low-urbanization areas can quickly and directly improve material use efficiency and reduce material costs, which matches the economic-oriented development concept of the region.

#### Closing” strategy in middle-level urbanization region

China’s average urbanization is 61.4%, with a growth rate of 2.42%/year^–1^. In the last 5 years, the influx of rural people into cities has slowed down as urban land becomes less available. In Beijing, the outflow of construction materials is mainly landfilling and recycling, of which 65% is landfilled disposal (1/3 being illegally landfilled and 2/3 being legally accepted) (Song 2015). But the total acceptance capacity of landfills is 39.2 Mt, and the annual landfilling of C&D waste in Beijing is 10.3 Mt, which means that the landfill space in Beijing will be exhausted in the next 5 years if the recycling rate is not improved (Song 2015; Chen and Liu 2021; Zhang et al. 2022). The major CEBM used for end-of-life construction materials management are recycling (closing) and reuse (slowing), but the possibility of reuse is limited by rudimentary demolition methods, unformed reuse markets, insufficient space for on-site sorting, etc. Currently, the recycling model based on closing strategies is the optimal option for CE implementation in the built environment in China.

#### “Slowing” strategy in high-level urbanization region

The case for slowing can be illustrated by the EU-25, a union of countries that includes a series of high-income countries located in Europe with an average urbanization of 75.5% and a growth rate of 0.41%/year^–1^. In contrast to the many short-lived buildings in countries undergoing rapid development, buildings in the EU-25 countries can last between 60 and 120 years, with an average demolition rate of 0.15% (Wiedenhofer et al. 2015). Because of their stable resident populations, these areas have little need for a massive expansion of the built environment to house new people. The dominant retrofit and maintenance activities lead to longer building life and material savings and limited waste generation compared to the building removal (Schiller 2007). An analysis of construction material stock sizes in EU-25 and their service lifetimes reveals the two most important factors driving material use necessary for renewal and maintenance (Wiedenhofer et al. 2015). The rule in EU-25 means that material consumption is most likely to be reduced in such countries through the stabilization of existing stocks and efforts to extend the service lifetime of buildings and infrastructure.

### Key findings and their implications

The results of this literature review suggest that spatial characteristics can significantly influence the dynamics of building materials and that more significant consideration of local spatial factors in construction activities to execute CE strategies (slowing, closing, and narrowing loops) and design CEBM can facilitate the CE transition in the built environment. Specifically, each strategy allows for material savings and optimization. The slowing strategy reduces natural material consumption and extends the material’s service life, the closing strategy reduces raw material consumption and waste generation, and the narrowing strategy reduces the demand for material and increases material use efficiency. Being influenced by multiple spatial features, the strategy can be prioritized to be applied to different types of regions for maximum effect, respectively.

Regions are divided into three categories of A (low), B (middle), and C (high) based on urbanization level, matching the three strategies of narrowing, closing, and slowing. The CEBM was developed based on the three strategies, respectively. But this does not mean that only a single CE strategy applies to a type of region. The hybrid forms of these strategies are possible, and sometimes strategies of slowing, closing, and narrowing resource loops are complementary. This study argues that the optimization effect on material dynamics can be maximized by prioritizing a matching strategy.

CEBM execution considers not only the maximization of effects such as material savings and environmental protection but also the supporting conditions of the models. As summarized by Lüdeke‐Freund et al. (2019), recycling involves multiple actors and the ability to deal with the specific physical and chemical properties of various materials, requiring a complex system to organize comprehensive reverse logistics that connects users, raw material suppliers, and component manufacturers. Refurbishment and remanufacturing require reverse and forward logistics and technical expertise on the product and how to refurbish or remanufacture them. Reuse and redistribution are based on providing access to used products, evaluating their market value, including slight enhancements or modifications, and creating a marketplace. These complex conditions are hardly met in areas where urban development is in an early stage (most of Africa and South Asia). Although the narrowing strategy proposed in this paper also needs some technical prerequisites to ensure material use efficiency, the technology based on the narrowing strategy starts from a modest base compared to the expensive mechanical equipment required for recycling, which only needs a locally tailored design to contribute obvious benefits.

While this study suggests that middle urbanized areas in the transitional phase match CEBMs based on closing strategies, it does not mean that narrowing and slowing can be ignored. Even though direct holistic reuse is a higher priority in the waste management hierarchy, recycling is still the best way to dispose of bulk materials since reusable components are only minor constituents of the demolished structure (such as windows and doors). Such places are in a state of urban renewal, between high-frequency new build activities and maintenance activities in the built environment, facing an increasing scarcity of land resources and materials. Recycling can reduce landfill occupation and save resources, but since the demolition, new build, and retrofitting in this type of region all contribute a certain percentage of material flow, we consider that narrowing and slowing cannot be neglected.

Highly urbanized areas, such as those in the EU-25, the USA, and Germany, are typically in a phase of stabilization, as seen through the primarily stable stock of materials and a focus on retrofitting and maintenance efforts (Schiller 2007; Wiedenhofer et al. 2015; Miatto et al. 2017). The shift towards retrofitting and maintenance and away from new build and demolition reduces negative impacts of natural resource mining and land consumption, which are significant concerns for highly urbanized areas.

### Limitation and strength of approach and next step

Methodologically, the approach of this study was based on a literature review, followed by the introduction of correlation analysis in the analysis of the cases. This approach has some similarities with causal analysis, but as causal relations are complex, not always easy to understand and a dispute with them is beyond the scope of this paper, we limit the analysis to correlations. The spatial features can be derived from each other without the consideration of chronological order. This correlation analysis helps organize spatial relationships, especially in grouping areas in an orderly pattern based on spatial characteristics. However, the relationships that are explicit and used can only represent general laws, and some subcategories with specificities are not included in these relationships. For instance, the relationship between terrain and density is generally positive, with gentle plains being livable and conducive to population aggregation and environmental construction. Arequipa is the second-largest city in Peru, with an average altitude of 2328 m. Despite the unfavorable topographic conditions, industrial development has contributed to the aggregation of the area due to its rich mineral resources (Mazer et al. 2020; Salmoral et al. 2020; Fraser 2021). The positive relationship between density and urbanization level is also only representative of the general situation, as some slum settlements do not reflect increased urbanization but are excluded from this study (Lilford et al. 2019; Ren et al. 2020).

The relationships between spatial features and CE strategies were compiled from case studies and are only applicable within the scope of review. Additional relationships may exist outside of the review’s framework. Despite limitations, the relationship networks presented in this study provide a general understanding of the built environment. Further research is needed to discover broader connections, and the rules outlined in this study have potential for expansion future studies that include more spatially informed case studies.

## Conclusion

Space as a factor influencing for shaping circularity in the built environment has rarely been the subject of research or directly specified in the CE research. This paper, based on a literature review, examines the role and effects of spatial information on transitioning the built environment to CE. A quantitative assessment of the literature was conducted, revealing a growth in research but uneven geographical distribution, with under-researched low-urbanized areas. Case studies were analyzed to provide specific examples of relationship between spatial information and CE in the built environment, highlighting the implicit connections in studies focusing on the dynamics and circularity of construction materials.

Different types of spatial features can influence the circularity of materials in the built environment by slowing (extending the lifespan of the product), closing (closing the cycle between end-of-use and production), and narrowing (reducing the consumption of materials) loops. The positive outcomes of implementing these CE strategies are not coincidental in most regions. Despite some uncertainties in the methods used to identify the network of relationships, the causal connections, when applied to the regional classification, accurately depict the three types of regions with distinct spatial characteristics. They align precisely the specific CE strategies.

The use of CE strategies, such as slowing, closing, and narrowing resource loops, through the implementation and design of CEBMs, can support the transition of the built environment to CE. By prioritizing the adoption of CEBMs in corresponding areas and synergistically combining other secondary business models, not only can economic advantages in material flows be gained but the urbanization process and the built environment can also be shaped with strong circularity.

Overall, improper adoption of CE strategies sometimes fails to achieve the circularity goals. Introducing spatial feature considerations into CEBMs design certainly contributes to resource and value circularity in the built environment. The findings are of particular interest to stakeholders (including actors in the design, construction, demolition, and recycling phases) seeking to circularize resources in the built environment to achieve environmental, economic, and social benefits. The study can provide insight into early work in various fields, including environmental engineering, industrial ecology, and sustainable management. While the results are drawn from cases in selected global regions, the implications for sustainability in construction and the challenges of urban resource shortages can apply to areas worldwide with similar geographic spatial characteristics.

## Data Availability

The data of the study are available on request.
